# Paludiculture can support biodiversity conservation in rewetted fen peatlands

**DOI:** 10.1038/s41598-023-44481-0

**Published:** 2023-10-23

**Authors:** H. R. Martens, K. Laage, M. Eickmanns, A. Drexler, V. Heinsohn, N. Wegner, C. Muster, M. Diekmann, E. Seeber, J. Kreyling, P. Michalik, F. Tanneberger

**Affiliations:** 1 University of Bremen, Bremen, Germany; 2 University of Greifswald, Greifswald, Germany

**Keywords:** Biodiversity, Wetlands ecology

## Abstract

Paludiculture, the productive use of wet or rewetted peatlands, offers an option for continued land use by farmers after rewetting formerly drained peatlands, while reducing the greenhouse gas emissions from peat soils. Biodiversity conservation may benefit, but research on how biodiversity responds to paludiculture is scarce. We conducted a multi-taxon study investigating vegetation, breeding bird and arthropod diversity at six rewetted fen sites dominated by *Carex* or *Typha* species. Sites were either unharvested, low- or high-intensity managed, and were located in Mecklenburg-Vorpommern in northeastern Germany. Biodiversity was estimated across the range of Hill numbers using the iNEXT package, and species were checked for Red List status. Here we show that paludiculture sites can provide biodiversity value even while not reflecting historic fen conditions; managed sites had high plant diversity, as well as Red Listed arthropods and breeding birds. Our study demonstrates that paludiculture has the potential to provide valuable habitat for species even while productive management of the land continues.

## Introduction

Peatlands contain massive stocks of carbon, storing over twice the amount of carbon in the biomass of all the world’s forests, despite covering only 3% of the Earth’s land surface^1^. However, these ecosystems have historically faced, and continue to face, enormous pressure and widespread degradation^2,3^. Once drained, peatlands emit substantial amounts of greenhouse gases (GHGs) through peat mineralisation and are currently responsible for approximately 5% of all anthropogenic GHG emissions^4^. Within Germany specifically, more than 95% of peatlands are degraded from drainage, with the majority being used for crops (21%) or meadows/pasture (60%), and this degradation contributes to 7% of Germany’s total GHG emissions^5,6^. Substantial further emissions from drained peatlands could be prevented by rewetting^7^.

While the need for rewetting is urgent, it is not possible to simply return all degraded peatlands into protected wilderness areas, as rural livelihoods are dependent on continued production from these areas^8^. Paludiculture—the productive use of wet or rewetted peatlands^9^—has been developed as a method for enabling rewetting while allowing farmers to continue working their land, though with an alternative land use. Paludiculture can take many forms, and in northeastern Germany can include harvesting common reed (*Phragmites australis*), sedges (*Carex* spp.), cattail (*Typha* spp.), or alder (*Alnus glutinosa*), and pasture with water buffalo (*Bubalus bubalis*)^8^. The biomass from these sites can be used for feedstock or biofuel^8^. Unlike conventional agriculture on drained peatland, paludiculture prioritizes preservation of the peat body^9^ and can contribute to the Paris Agreement targets (warming below 2 °C) through reduced GHG emissions^8,10,11^. To preserve the peat body and allow for carbon sequestration, specialized mowing equipment adapted to wet conditions is used, and water levels are kept at or above ground level year-round^8^. Deeply drained peatlands are especially good candidates for paludiculture, as they are unlikely to return to a historic state even after restoration^9,12^. Continued production on this land is an equitable approach, enabling farmers to remain on the land, and local communities to steward their own peatland resources^1,13^.

Peatland degradation has resulted in substantial loss of biodiversity^14^. Fens in particular have lost biodiversity due to a reduction of traditional management, both from abandonment and intensification of agriculture through drainage and eutrophication^15,16^. Peatlands with a history of agricultural use have become adapted to regular disturbance, leading to declines in biodiversity when management is abandoned^15^. Biodiversity loss may occur from eutrophication in drained and rewetted peatlands due to past agricultural use and the mineralization of peat^13^. In these cases, mowing of fens may be essential for reducing eutrophication and maintaining biodiversity^17,18^. Without mowing or other forms of management, rewetted fens may be dominated by a few tall and competitive species, resulting in a loss of low growing plants, rare species, and those with a low competitive ability^14,19–21^. Paludiculture sites are likely to have greater fen biodiversity and more wetland species compared to their drained state^22^. Even agricultural or open landscape species may benefit from peatland rewetting and management due to the subsequent opening of vegetation structure^16,22^.

There is a need to understand how biodiversity responds to paludiculture and how to maximize outcomes for biodiversity conservation. Rewetted peatlands have been found to create novel ecosystems that differ in their plant and spider biodiversity compared to historical peatlands^12,23^. Especially lacking is an understanding of the response of biodiversity to different intensity levels of paludiculture^22^. In this study, we assessed the biodiversity of plants, breeding birds, carabid beetles and spiders using both quantitative and qualitative methods. Six sites located in northeastern Germany were studied in 2021 and 2022. These sites varied in their dominant vegetation type, either *Carex* or *Typha* species, and in their land use intensity, either unmown, mown occasionally, or mown annually. Biodiversity was compared across sites to assess quantitative diversity and Red List status was used to assess qualitative diversity values. We demonstrated that paludiculture sites can host high vegetation diversity and critically endangered breeding birds, as well as spiders and carabids of conservation concern. Each taxon is expected to respond differently to management, indicating the need for a multi-taxon perspective to understand the impact of paludiculture on the biodiversity of rewetted peatlands.

## Results

A total of 78 plant, 18 breeding bird, 55 carabid, and 73 spider species were identified. A total of 32 Red Listed species (3 plants, 7 birds, 12 carabids, and 10 spiders) were present; all but three of these (spiders) occurred in managed peatlands. Most Red List species present were those associated with wetlands (28), or open landscapes (3 breeding birds). *Carex* sites generally had higher mean vegetation coverage than *Typha* sites; sites ranged from 80-100% mean coverage to 60–80%, respectively. Trees and shrubs were almost never present, and bryophytes were only occasionally encountered. Litter cover was generally high (> 85%) except for the high intensity *Typha* site which had minimal litter. A full species list is available as a supplementary file.

### Quantitative analysis

**Figure 1:**
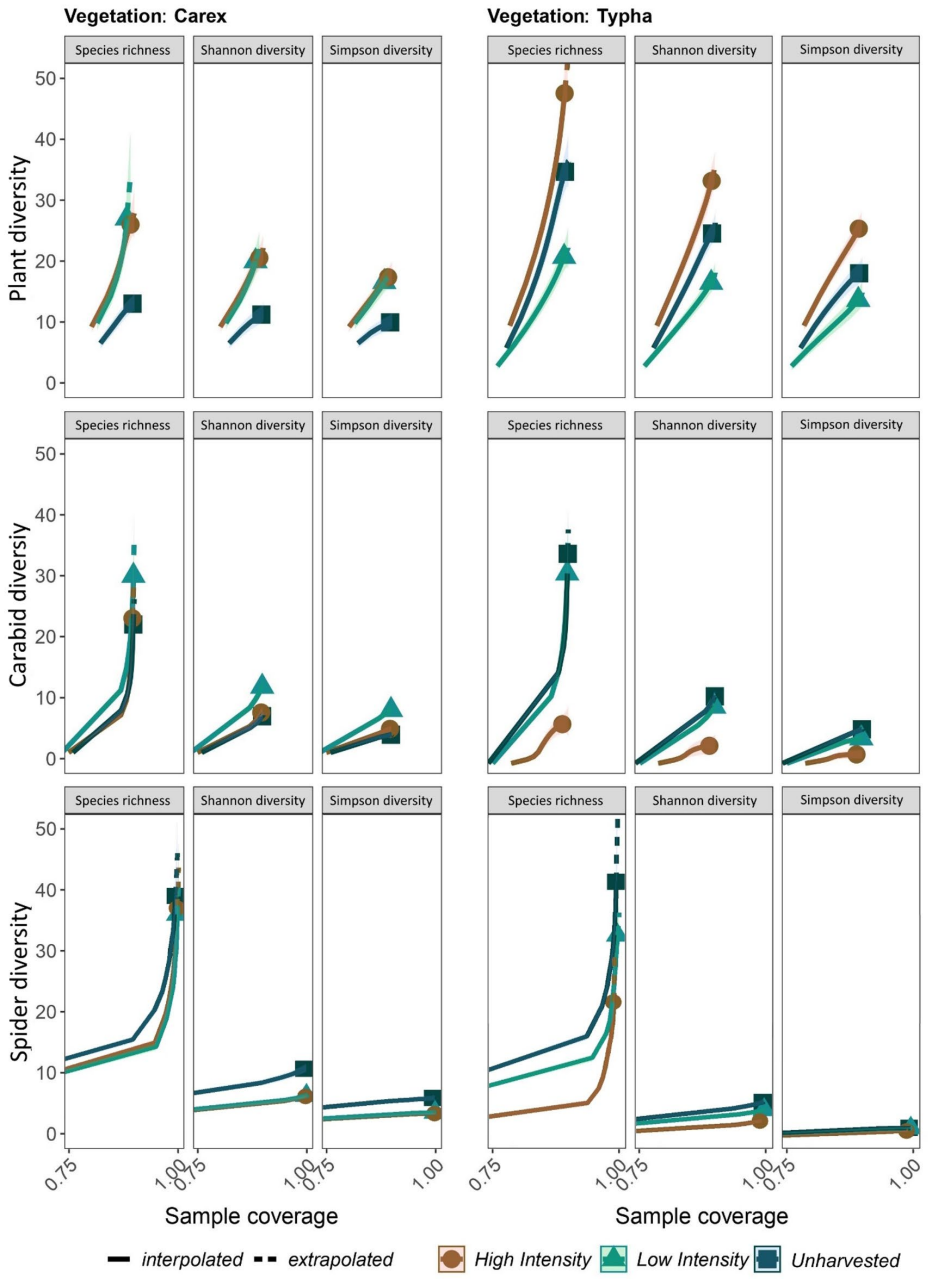
Coverage based biodiversity extrapolations for different taxa comparing paludiculture intensities for Carex and Typha as target species. Estimate of sample completeness is given as sample coverage which is used to standardize samples according to the iNEXT.4 package. Diversity results are extrapolated and interpolated across the range of Hill numbers^24^. Thus, diversity at each site is compared using species richness, which is biased towards rare species, Shannon diversity, biased towards common species, and Simpson’s diversity, biased towards dominant species. Sites are compared at equal sample coverage, given as the coverage maximum (double the smallest sample size), where a sample coverage of 1.0 for Simpson’s diversity indicates 100% of dominant species are predicted to have been found^24^. Here, vegetation is compared at a maximum coverage of 0.95, and carabids and spiders both at 0.99. Bird results are not provided due to insufficient coverage (coverage maximum of 0.75). Shown are 83.4% confidence intervals, whose non-overlap indicates a significant difference at alpha = 0.05^25–27^.

The iNEXT package, developed by Chao et al.^24^, was selected for the quantitative analysis because it both quantifies sample completeness and provides diversity estimates across the range of Hill numbers. Sample coverage values, which are a measure of sample completeness, were generally close enough to 1.0 (or 100% complete) to enable interpretation of iNEXT results, except for breeding birds. The newly developed high intensity *Typha* cropping site had significantly higher predicted plant diversity across the range of Hill numbers, while the low intensity *Typha* site had significantly lower diversity. The managed *Carex* sites had significantly more plant species than the unmanaged site (Fig. 1). Results for breeding birds generally showed insufficient sample coverage for interpretation (coverage maximum 0.75). The high intensity *Typha* site had significantly fewer carabid species: the site had one third of the estimated species richness of any other site. The spiders in the unharvested *Carex* site had around 60% higher Shannon and Simpsons diversity than other *Carex* sites, and higher species richness in the unharvested *Typha* site. All other sites were similar in their quantity of spider species. Vegetation and spiders responded oppositely to management; plant diversity generally increased in mown sites, but spider diversity decreased.

### Qualitative analysis

Across all sites, most of the species identified were typical for wetlands (74%). Sites did not reflect a historic mire state since they had few mire-specific species. Species of conservation concern were found from all taxa; the species of greatest concern and mire-specific species have been listed (Table 1)^28–31^. Additionally, thirteen threatened species and eight near threatened species were present at managed sites (complete list of Red List species available as a supplementary file).

**Table 1:** List of species of conservation concern recorded at the study sites.

Conservation status	Mire-specific	International red list	German red list: threatened with extinction	German red list: highly threatened
*Carex*-unharvested	*▼ Carorita limnae*a *▼ Diplocephalus dentatus* *▼ Pirata piscatorius*			*▼ Carorita limnaea* *▼ Centromerus semiater* *▼ Diplocephalus dentatus* ♦* Locustella naevia*
*Carex*-low intensity	*● Triglochin palustris* *▼ Carorita limnea* *▼Pirata piscatorius*	*▼ Dolomedes plantarius*	♦* Gallinago gallinago*	█* Elaphrus uliginosus* *▼ Carorita limnaea* *▼ Dolomedes plantarius*
*Carex*-high intensity	*▼ Pirata piscatorius*		♦* Gallinago gallinago*	
*Typha*-unharvested	*▼ Pirata piscatorius*			*▼ Diplocephalus dentatus* ♦* Locustella naevia* ♦* Saxicola rubetra*
*Typha*-low intensity	*▼ Pirata piscatorius*	♦* Vanellus vanellus*		█* Elaphrus uliginosus* ♦* Anthus pratensis* ♦* Locustella naevia* ♦* Saxicola rubetra*
*Typha*-high intensity	*● Juncus subnodulus* *▼ Pirata piscatorius*	♦* Vanellus vanellus*		♦* Saxicola rubetra*

Mire-specific species, IUCN Red List species, and the top two categories of the German Red List have been included. Taxa are indicated by the symbol: plants ●, carabids █, spiders ▼, birds ♦.

## Discussion

Quantitative analysis showed no consistent diversity response to the intensity of use of rewetted fen peatlands, regardless of dominant vegetation type. Qualitative results demonstrated that all sites, and, consequently, all land use intensity levels, were providing habitat for Red List wetland species. Given that intensive grassland on drained peatlands does not provide habitat for fen communities^32^, our findings underline that paludiculture can support fen biodiversity and conservation better than a drained state. Additionally, management supported higher vegetation diversity then an unharvested wet state. However, birds, arthropods, and plants all varied in their biodiversity between sites and management intensity, thus supporting the need for variation of land use intensity in the landscape, as also suggested by other studies^33^.

### Quantitative analysis

Managed *Carex* sites all had similarly high vegetation diversity values. In contrast, the unharvested *Carex* site had significantly lower diversity and had highly uniform and tall vegetation. Tall vegetation can restrict the growth of light-dependent species in fens^34,35^. This study, like others, found that mown sites have the capacity to host higher plant species richness than unmown sites^34,36–41^. Despite its isolated location and recent rewetting, the high-intensity *Typha* site had significantly higher diversity then other sites. However, given the site was recently established (2019), species diversity may change over time. *Typha-*low had the lowest diversity values, which may be attributed in part due to the high proportion of ruderal plant species (*Urtica dioica, Cirsium arvense*) compared to other sites.

The high intensity *Typha* site had significantly lower carabid diversity than all other sites. A contributing factor may be the low willingness of carabid specialist species to cross unfavorable terrain, reducing the chance to disperse to new areas^42^. This site was rewetted only two years before our observations and is a hydrologically isolated fen in a landscape dominated by drained peatlands used as pasture. The other sites that were studied had been rewetted around twenty years prior (Table 1). A study of a *Sphagnum* paludiculture site found that during the first three years after rewetting, spider community structure changed considerably, but after three years the overall community structure remained stable^43^. To better support carabid species, connectivity to other peatlands should be restored^42^, and it may take time for stable populations to form. Species re-introduction may be helpful and has been used for example in the partially successful reintroduction of the fen raft spider (*Dolomedes plantarius*) in the UK^44^. However, the presence of rare and threatened species in the study sites indicates that species assemblages are establishing in a positive trajectory. Results from the high intensity site vary between all groups and show both significantly more plant and less carabid beetle diversity than all other sites; diverging diversity values between carabids and plants were also found by Görn & Fischer^45^ emphasizing the importance of multi-taxon studies.

Spider diversity results were unique compared to other taxa, as the unharvested *Carex* site had significantly higher Shannon and Simpson’s diversity than all other sites. Plants and carabids had moderate to very low diversity values for this site. Studies on spiders in fens have found that mowing reduces litter and vertical vegetation, and thus may reduce structure-dependent species like rare wetland spiders and some widespread species^46,47^. Research on other invertebrate groups also found lowest species richness in recently mown reedbeds^33^. These factors may be contributing to high diversity values in the site without management. Higher diversity of spider and bird species than carabids at the high intensity *Typha* site may relate to mobility, since some spiders have “ballooning” capabilities and thus higher dispersal ability^43^.

### Qualitative analysis

All sites had a high proportion of mire-typical and general wetland species which aligns with work by Tanneberger et al.^22^, who found that paludiculture sites host primarily species adapted to wet environments. However, sites lacked indicators of a natural mire, since very few mire-specific species were identified. Rewetted peatlands have been found to differ in their plant diversity, hydrology, and geochemistry compared to near-natural peatlands^12^. These rewetted landscapes typically have tall graminoid plants, are eutrophic, and have a higher water table^12^. Despite its recent rewetting and isolated location, the high intensity *Typha* site hosts Red List species from all studied taxa. For example, northern lapwing populations have declined dramatically in the last thirty years as their habitat has decreased from both intensification and abandonment of land use and may benefit from low or moderate management intensity^16,48–50^. Moreover, multiple bird species associated with landscapes other than wetlands, including agricultural (*Emberiza calandra*) or open landscapes (*Saxicola rubicola*, *Saxicola rubetra*), were breeding in the paludiculture sites indicating that such sites can indeed host at-risk species. This is in accordance with other paludiculture projects^43^. While in restored fens it may be preferable to have a high number of mire-specific species, this may not be the case for paludiculture sites. For example, if paludiculture sites can provide habitat for endangered agricultural and open landscape species whose habitat is disappearing, this may also be considered a positive effect of such land use.

Further research over multiple years and on many more sites is needed to understand the conservation and biodiversity value of paludiculture as sites change. For example, a study by Valkama et al.^38^ showed that after several years, mowing significantly decreased invertebrate abundance, but in the short-term (1–2 years) the sites appeared unaffected^34^. A study by Muster et al. on a *Sphagnum* paludiculture site noted that each successional stage had different species, and even at early stages sites had high conservation value species, but not mire-typical species^43^. In our study, all but the high intensity *Typha* site reflect a long-term state, since rewetting occurred in the early 2000s (Table 2). Future work on paludiculture biodiversity should study multiple animal groups, as each may respond differently to management, and additionally, more multi-year studies are important to understand succession, annual fluctuations, and dispersal in newly established sites or according to mowing regime. Long term monitoring of such paludiculture sites would provide more information on typical species and conservation value at each successional stage, especially on sites that are not mown annually (low-intensity management), where species composition may vary temporally. Many factors influence the impact of mowing on biodiversity, including the block size in when creating a mosaic of mowing regimes^47^, mowing technique and machinery^51^, and time of year^49^. More sites and thus spatial replication are needed for a robust understanding of how these factors influence diversity at paludiculture sites.

**Table 2:** Site descriptions, use, and history.

Name	Mowing intensity	Area (ha)	Year drained	Year Rewetted	Water level class	pH	Mean vegetation height (cm)	Vegetation height SD (cm)	Water level amplitude (cm)
*Carex-*unharvested	None	1.0	1925	2002	5+	9.3	92.5	8.2	62.7
*Carex*-low intensity	Infrequent	3.5	1925	2002	5+	8.7	80.0	8.6	57.2
*Carex*-high intensity	Annual	2.5	1925	2002	5+/6+	9.5	75.0	25.2	57.1
*Typha*-unharvested	None	16.5	1967	2005	4+/5+	8.9	120.0	40.6	61.1
*Typha*-low intensity	Infrequent	5.8	1940	2001	5+	8.5	90.0	21.5	11.0
*Typha*-high intensity	Annual	9.0	1935	2019	6+	8.8	82.5	55.8	20.0

Water level class calculated from summer 2021 and winter 2021/22 median water level based on water level classification from Couwenberg^55^, adapted from Koska^56^. Water level of 4+ may preserve peat (depending), while levels of 5+ and 6+ are peat preserving or even peat forming^57^. Mean vegetation height was taken as an average across the entire site, all other values are from a single point at the site in 2022. Amplitude gives the difference between the minimum and maximum water level during the recorded period. pH data was collected in 2021.

## Methods

### Site selection

**Figure 2:**
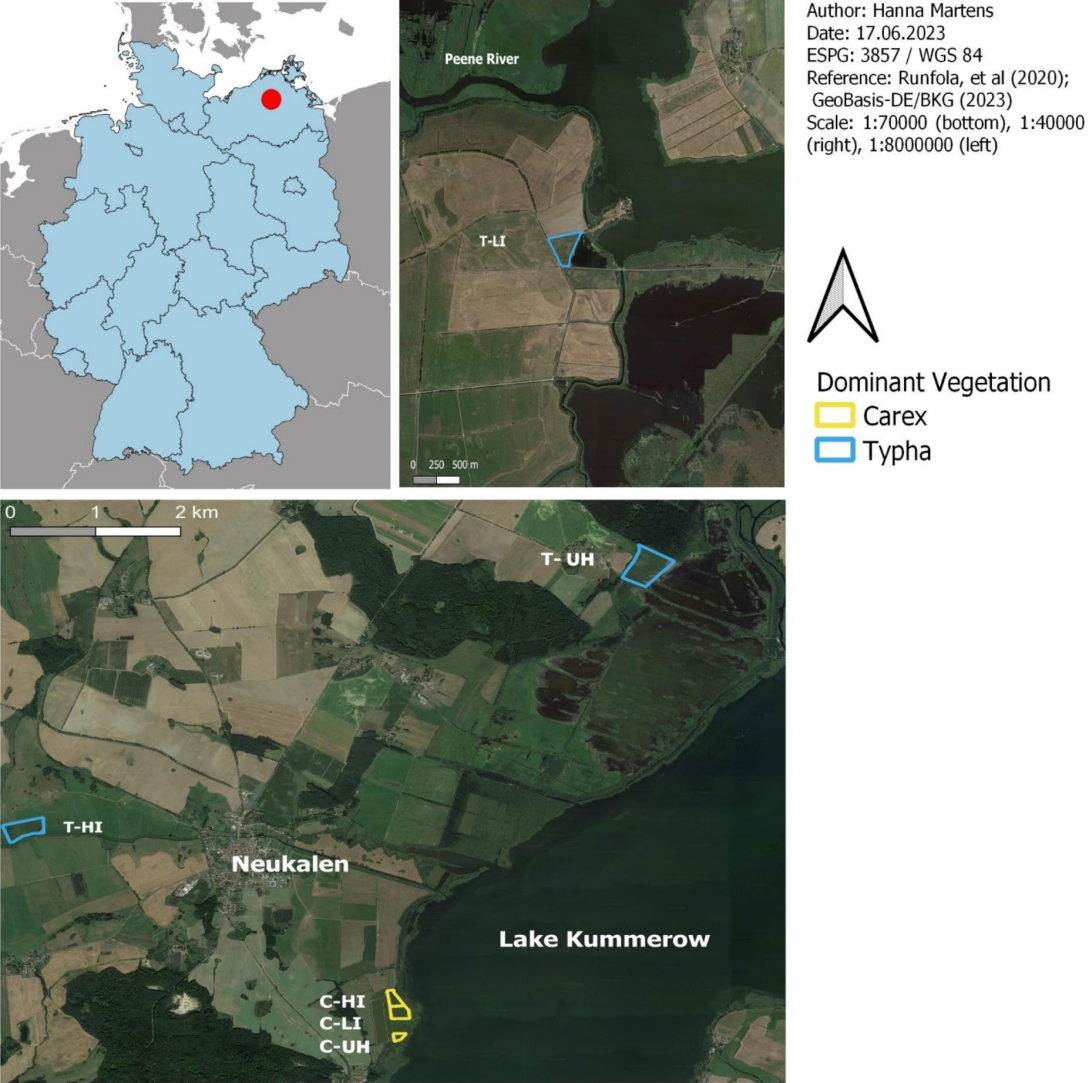
Map of sites in Macklenburg-Vorpommern, Germany^58,59^. Sites are labelled by their dominant vegetation type, Carex (C), or Typha (T), and the land use, including unharvested (UH), low intensity (LI), and high intensity (HI). The majority of sites were located near Neukalen but the high intensity Typha site (T-LI) was located approximately 70 km east near Anklam.

The study sites are in the state of Mecklenburg-Vorpommern in northeast Germany (Fig. 1, Table 1). Site boundaries were delineated by barriers (roads, open water bodies, ditches) or by transition to a new mowing regime or vegetation type. Sites were selected for their vegetation type, either *Carex* or *Typha*, and had dominant species of either *Typha latifolia*, or *Carex acuta, C. acutiformis, and C. disticha.* All sites have a history of deep drainage and subsequent rewetting in the early 2000s as permanent grassland paludiculture^52^, except for the high intensity *Typha* site, which was rewetted in 2019 and developed as a cropping paludiculture site with planted *Typha*. The study locations varied in their connectivity with surrounding natural fen habitat; the *Carex* sites are all three similarly close to peatlands that were only slightly drained (north of Neukalen and on the eastern side of Lake Kummerower) (Fig. 2), *Typha-*unharvested and *Typha-*low were surrounded partly by agriculture and partly by other rewetted peatlands, and the *Typha-*high site was isolated, surrounded by drained peatland used as grassland and the Teterower Peene river, and rewetted in 2019 (Table 2). High intensity sites were harvested completely every year, and low intensity sites were mown every two to three years, in some years only mulched (without biomass removal). The sites are in a temperate climate and experience a mean temperature of 9.5 °C, with around 735 mm of annual precipitation, with most of this falling in the summer months^52^. Site selection was limited since few paludiculture areas have been established thus far and more replicates were not readily available, especially for managed sites. Additionally, further sampling would have demanded too many resources and would have been beyond the scope of the current study. Therefore, our study had replicates within each site, but did not have true replicates for management intensity. However, geostatistical analysis of fen peatlands has demonstrated that spatial autocorrelation is rarely present^53,54^. This suggests that the spatial replicates within each of our six sites can be treated as independent and their variation is representative for their respective vegetation type.

### Data collection

Vegetation data was collected in 2022, and breeding bird, carabid, and spider data in 2021. Water level classification is based on water level measurements taken at a representative permanent monitoring well located at each site measured from April 2021 until February 2022. Water levels are classified based on Couwenberg^55^, adapted from Koska^56^.

Vegetation was surveyed in late June and early July of 2022. Plots were placed using stratified random sampling and number of plots varied due to differences in the size of each site (Table 2) (*Carex*-unharvested: 6, *Carex*-low and high: 10, *Typha*-unharvested: 20, *Typha*-low: 18, *Typha-*high:22).Two by two-metre plots were placed at regular intervals along a transect running through the site center. Additional plots were placed at random if multiple vegetation zones were present. Edges, open water, and areas heavily trampled by mowing near site entrances were avoided, resulting in a small reduction in sampling area. Cover values of each species were estimated as percent coverage at < 1% coverage and intervals of 10%. These values were then converted into presence-absence data to fit the format required by the iNEXT package. Species were identified using Streeter et al.^60^ and names verified using Euro + Med PlantBase^61^.

Breeding birds were surveyed following the breeding bird territory survey method outlined by Südbeck et al.^62^. Surveys were conducted over five mornings starting 30 min before sunrise and two evenings starting 30 min after sunset. All birds singing, calling, and all those engaged in behavior indicating breeding within the site were recorded using QField and mapped using QGIS. Breeding pairs were determined based on their behaviour and the time of year^62^. Surveys were conducted at the end of March, end of April, middle of May (one evening, one morning), beginning of June (evening survey), middle and end of June. Sites were surveyed over three days each time, always with a minimum of seven days between each survey round. The order of sites surveyed, and the route taken while surveying was altered each time.

Carabid beetles and spiders were collected using pitfall traps (six per site) and additional floating traps were placed at the three *Typha* sites to collect arthropods due to high water level. Pitfall traps were made from a standardized colorless transparent reusable plastic cup^63^. Cups were held in place using tent pegs. Floating traps were constructed using a cup surrounded by a Styrofoam ring and were weighted to keep the cup rim at surface level^64^. These were set within a polypropylene pipe, diameter of 15 cm and length of 100 cm to hold traps in place. Each pipe had several 5 cm diameter holes to allow arthropods to enter and was plugged on the upper end to prevent rainwater and debris from entering. Sampling cups had a diameter of 8 cm, depth of 10 cm, and contained a solution of ethanol, water, glycerin, and acetic acid at a ratio of 4:3:2:1 and unscented soap^65^. Locations of traps were recorded with GPS and marked with bamboo sticks and were spaced 10 m apart and at least 20 m away from site boundaries. Five sampling periods occurred in spring (April–June) and three in autumn (September and October) for a total of eight. Each sampling period lasted 14 days. Identification for carabids was done following Müller-Motzfeld^66^ and nomenclature using Schmidt et al.^67^ . Spider identification and nomenclature followed Nentwig et al.^68^.

### Data analysis

General analysis was done in R^69^ using RStudio^70^ and the package tidyverse^71^ and visualization done using viridis^72^, ggrepel^73^, gt^74^, MetBrewer^75^, and ggplot2^76^. Several methods of biodiversity analysis were utilized, given that no one method has been found to be entirely effective or representative of site diversity. Quantitative biodiversity analysis was made using iNEXT^77,78^, iNEXT.4steps^79^, and devtools^80^. The iNEXT package provides diversity estimates across the range of Hill numbers and thus across the range of sensitivity to species abundance and was used following Chao et al.^24^. The method is based on the work of Hill^81^ who found that species richness, Simpson’s diversity and Shannon’s diversity can be placed on a continuum of diversity measures based of their bias towards rare species. This continuum approach is more robust than using any of these diversity estimates individually since each are biased and when used alone may provide contrasting results^24,82,83^. iNEXT method enables comparison using sample completeness rather than sample size, allowing for comparison between differed sized sites without having to reduce to the smallest sample size for comparison^24,84^. The method for sample completeness estimation is formulated on the codebreaking work of Allan Turing during WWII and estimates the amount of information that is unknown to quantify what is known, given the frequency that something appears exactly once or exactly twice^84^. The iNEXT.4steps package provides analysis in four steps, as suggested by the name, but only two of these were utilized for this analysis. Sample coverage (step 1) and non-asymptotic coverage-based rarefaction and extrapolation (step 3) were the focus, since they provide analysis of sites with uneven sampling intensity. Step two (asymptotic and empirical diversity) has been left out, since samples were insufficiently complete to detect true diversity, and step four (evenness) was also omitted, since a lack of replicates resulted in large and inconclusive confidence intervals^24^. Samples were bootstrapped 50 times (the packages default) to estimate 83.4% confidence limits which were used to determine significance of differences between the land use intensities. Confidence intervals were set based on research that demonstrates non-overlap of 83.4% confidence limits correspond with approximately an alpha of 5%^26,27^.

Species were also evaluated qualitatively, both concerning their endangerment status and their typical habitat preference using literature for northeast Germany. Mire-specific plant species were identified using Hammerich et al.^85^ and mire-specific spider species using Martin^86^. Furthermore, area-specific literature was used to determine the typical habitat for each species (vegetation^60,87^, breeding birds^88–90^, carabids^91^, and spiders^92,93^). The goal of this classification was to determine if paludiculture sites were attracting wetland species, or if the sites continue to host mostly species associated with traditional agricultural land, generalists, or other habitat types. National level Red List information was obtained from the German Red List Center for plants^94^, birds^31,95^, carabids^96^, and spiders^28^. International information comes from the IUCN Red List website^97^.

## Conclusion

The approaches taken in this study provide a multi-taxon view of biodiversity in the selected paludiculture sites by using four different taxa and both a qualitative and quantitative approach for assessing biodiversity. All sites, irrespective of management intensity, hosted species with high national and international conservation value, indicating that not only protected “wilderness” sites but also paludiculture sites can provide refuge for endangered species. However, these sites did not resemble natural fen conditions and had few mire-specific species but did contain primarily wetland species. The site with greatest management influence (*Typha-*high intensity) had both the lowest and the highest qualitative biodiversity values depending on the taxon. Thus, further research is needed to understand long-term biodiversity trends in these novel ecosystems, and many more sites should be established and studied to create a more robust understanding of the factors shaping biodiversity in paludiculture sites. Since responses varied between taxa, management should aim to provide a habitat mosaic with variation in management intensity. Also from a biodiversity perspective, efforts towards rewetting and management of degraded peatlands should continue, since it has been demonstrated that this land use supports high biodiversity and species quality compared to a drained peatland.

### Supplementary Information

Supplementary Information.

## Data Availability

All data generated or analysed during this study are included in the supplementary information files of this published article.
